# Supplementary material for: Ultraprecise Detection of Influenza Virus by Antibody-Modified Graphene Transistors
Source: Sensors (Basel). 2025 Feb 5;25(3):959. doi: 10.3390/s25030959 (PMC11820836; doi:10.3390/s25030959)
Supplement: Supplementary file 1 [file sensors-25-00959-s001.zip › sensors-3409735-supplementary.pdf]

Supplementary Information

Limit of detection (LoD)

The LoD can be obtained via Eq. (1):

$$LoD = \frac{3\sigma}{S} \tag{1}$$

where σ is the standard deviation of the testing response to the negative control and S is the sensitivity, which is acquired as follows:[4]

$$S = \frac{x' - x_0}{c' - 0} \tag{2}$$

where $\boldsymbol{c'}$ is the tested concentration, $\boldsymbol{x'}$ is the response corresponding to $\boldsymbol{c'}$, and $\boldsymbol{x_0}$ is the response corresponding to the negative control.

Supplementary Tables

Table S1. Baseline characteristics of 106 clinical influenza A samples from nasopharyngeal swabs.

Variables	**Overall**	**Confirmed Patients**	**Non Confirmed**
**Sex**			
Female	47	15	32
Male	59	17	42
Age, years, mean (SD)	17 (15)		
Fever (>38.5 °C)	29	25	4
Mean of date of specimen collection after onset of symptom (range)	3 (1–5)		
Chest X-ray suggestive of Influenza A infection	51	32	19
**Etiological diagnosis**			
Positive on PCR	36	32	4
Positive stool on Ab-GFET	18	18	0

Table S2. Ct values measured by RT-qPCR for influenza A-positive samples.

Sample	Ct value	Sample	Ct value	Sample	Ct value
P1	37.99	P12	26.13	P23	25.75
P2	36.08	P13	19.39	P24	36.61
P3	35.5	P14	23.06	P25	33.61
P4	37.21	P15	28.49	P26	33.58
P5	26.18	P16	30.76	P27	33.55
P6	21.92	P17	30.46	P28	30.53
P7	24.95	P18	28.42	P29	28.77
P8	25.9	P19	27.17	P30	39.89
P9	22.94	P20	31.33	P31	28.18
P10	23.15	P21	23.5	P32	22.94
P11	38.04	P22	25.6		

Table S3. Comparison of existing diagnostic methods.

Detection method	Analyte type	Sample-to-answer time (min)	AUC (%)	Sample size	Power (%) *	Effect size**	Ref
CRISPR-Cas13a	HIV	40	92.2	158	100	0.25	[1]
Quantitative PCR	HRV	360	77−84	178	100	0.24	[2]
BEETLES[2]	SARS-CoV-2	3	91.9	62	89.7	0.39	[3]
Bioscreen	SARS-CoV-2	21	99.2	37	72	0.48	[4]
FET	SARS-CoV-2	35	92	30	64	0.52	[5]
LAMP	SARS-CoV-2	30–50	80−85	40	75	0.47	[6]
LEAD	SARS-CoV-2	6.5	87.4	103	98.3	0.31	[7]
Nanoplasmonic assay	SARS-CoV-2	90	87	60	89	0.40	[8]
Thermophoretic assay	SARS-CoV-2	15	100	51	83.8	0.42	[9]
SERS-LFA assay	Influenza A	>1200	84.6	39	74	0.47	[10]
SERS	Influenza A	30	95.5	52	84	0.5	[11]
MEIA	Influenza A	15	94.6	79	78	N/A	[12]
Au NP	Influenza A	30	96.16	33	N/A	N/A	[13]
FICT	Influenza A	25	91	34	55.88	N/A	[14]
QD-FICT	Influenza A	25	93.75	48	79.17	0.5	[15]
CRISPR/Cas13a	Influenza A	195	83.9	N/A	N/A	N/A	[16]
CRISPR/Cas13a	Influenza A	180	98	33	92	0.5	[17]
NPAb-GFET	Influenza A	1.2	99.1	106	100%	0.4	This work

Note: N/A, not available. BEETLES[2], bioengineered enrichment tools for lateral flow assays (LFAs) with enhanced sensitivity and specificity. FET, field-effect transistor. LAMP, loop-mediated isothermal amplification. LEAD, low-cost electrochemical advanced diagnostic. Au NP, gold nanoparticle. PCR, polymerase chain reaction. SERS, surface-enhanced Raman scattering. MEIA, multichannel electrochemical immunoassayplatform. FICT, fluorescent immunochromatographic strip test. QD-FICT, quantum dot fluorescent immunochromatographic strip test.

* The efficacy analysis was carried out by one-way ANOVA with effect size = 0.35 and $\alpha = 0.05$.

** The efficacy analysis was carried out by one-way ANOVA with Power = 95% and $\alpha = 0.05$.

Supplementary Figures

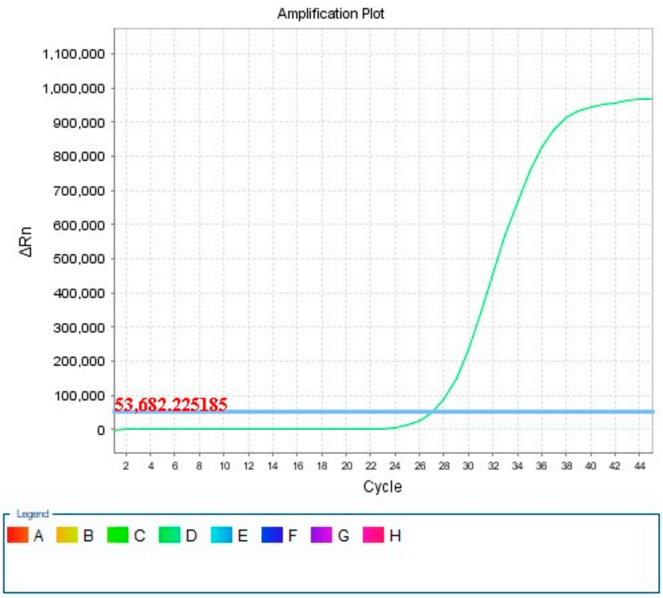

Figure S1. Representative amplification curve graphs for the influenza A nucleic acid assay are from patient 5, the original image exported from the ABI7500 amplifier.

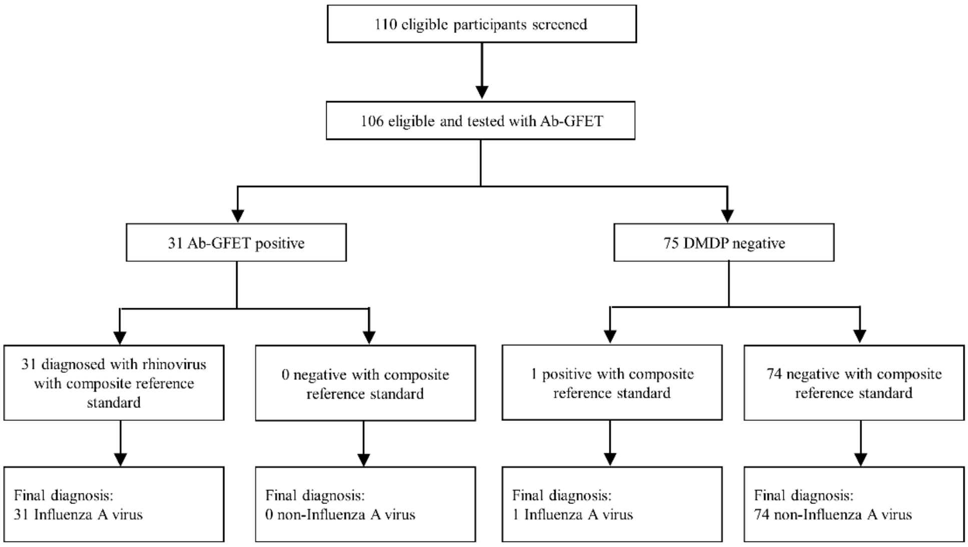

Figure S2. Trial profile for influenza A detection. The diagnostic performance of NPAb-GFETs is compared with that of PCR, which is set as the composite reference standard in the study.

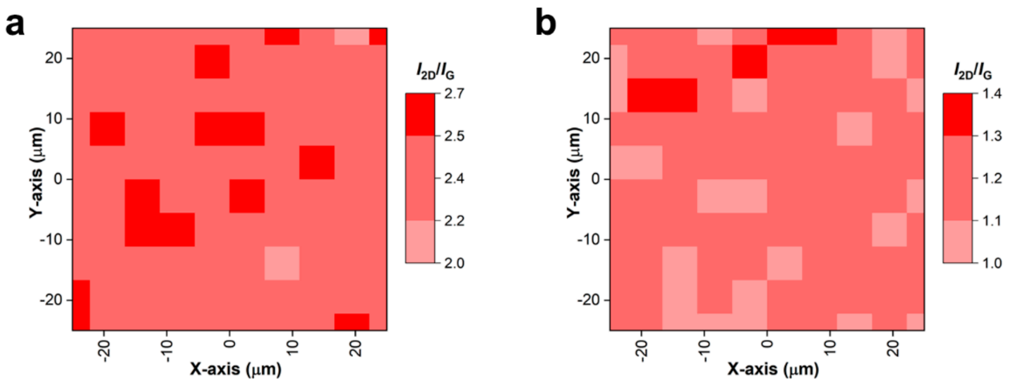

Figure S3. Characterization of graphene. I_{2D}/I_G mapping image of graphene before antibody modification (a) and I_{2D}/I_G mapping image of graphene after modification (b).

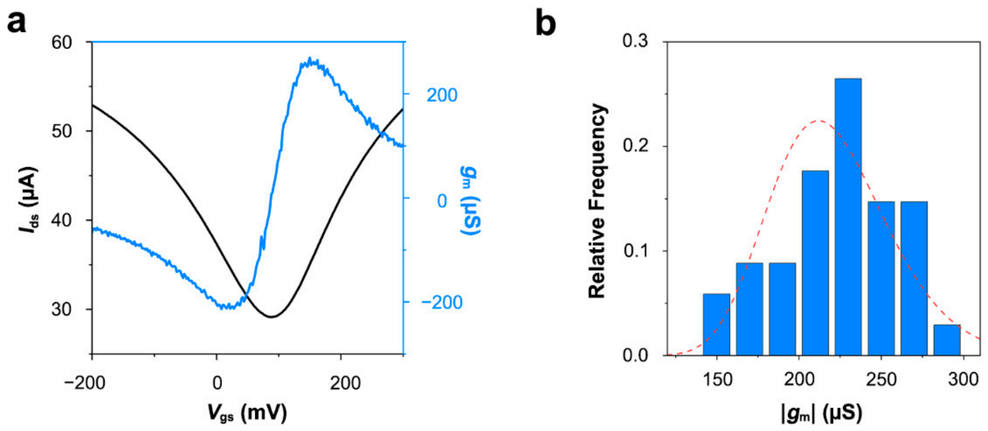

Figure S4. Device Uniformity of GFET Biosensors. (a) The I_{ds}–V_{gs} transfer characteristic curve of the GFET biosensor and its first-order differential curve; (b) The frequency distribution statistical results of graphene transconductance.

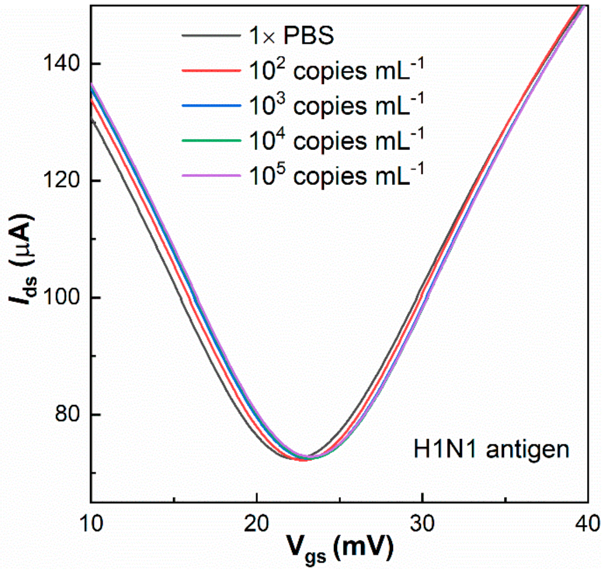

Figure S5. I_{ds} versus V_{gs} curves upon the addition of H1N1 antigen in 1× PBS.

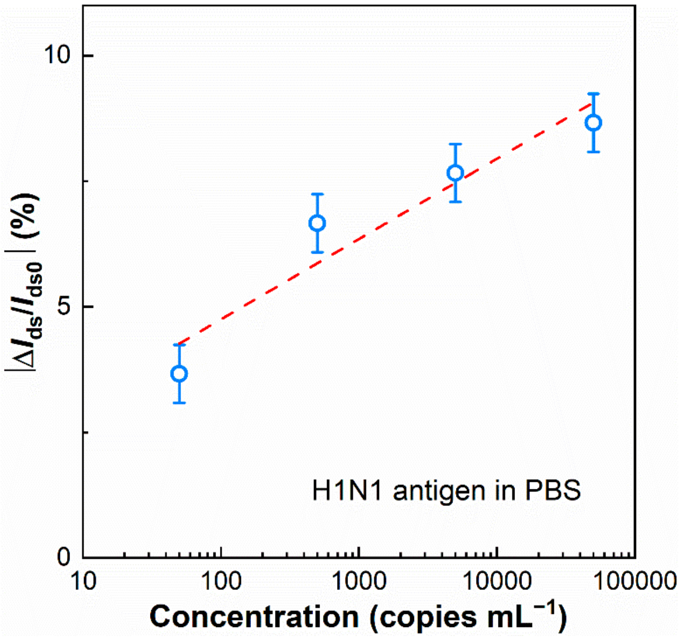

Figure S6. Detection of H1N1 analytes by NPAb-GFETs. The error bars are determined by the standard deviation of 3 measurements, which denote the median ± 95% CI.

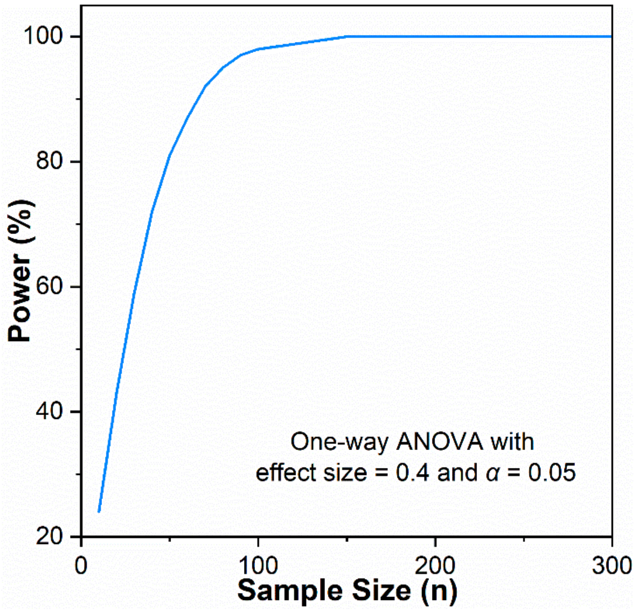

Figure S7. Power versus sample size curve. The statistical power increases little when the sample size is larger than 100. Thus, the sample size is appropriate in this study.

Supplementary references

1. Li, X.; Su, B.; Yang, L.; Kou, Z.; Wu, H.; Zhang, T.; Liu, L.; Han, Y.; Niu, M.; Sun, Y.; Li, H.; Jiang, T., Highly sensitive and rapid point-of-care testing for HIV-1 infection based on CRISPR-Cas13a system. *BMC Infect Dis* **2023,** 23, (1), 627.
2. van der Schee, M. P.; Hashimoto, S.; Schuurman, A. C.; van Driel, J. S.; Adriaens, N.; van Amelsfoort, R. M.; Snoeren, T.; Regenboog, M.; Sprikkelman, A. B.; Haarman, E. G.; van Aalderen, W. M.; Sterk, P. J., Altered exhaled biomarker profiles in children during and after rhinovirus-induced wheeze. *Eur Respir J* **2015,** 45, (2), 440-8.
3. Horii, Y.; Matsuda, S.; Toyota, C.; Morinaga, T.; Nakaya, T.; Tsuchiya, S.; Ohmuraya, M.; Hironaka, T.; Yoshiki, R.; Kasai, K.; Yamauchi, Y.; Takizawa, N.; Nagasaka, A.; Tanaka, A.; Kosako, H.; Nakaya, M., VGLL3 is a mechanosensitive protein that promotes cardiac fibrosis through liquid-liquid phase separation. *Nat Commun* **2023,** 14, (1), 550.
4. Macchia, E.; Kovacs-Vajna, Z. M.; Loconsole, D.; Sarcina, L.; Redolfi, M.; Chironna, M.; Torricelli, F.; Torsi, L., A handheld intelligent single-molecule binary bioelectronic system for fast and reliable immunometric point-of-care testing. *Sci Adv* **2022,** 8, (27), eabo0881.
5. Li, J.; Wu, D.; Yu, Y.; Li, T.; Li, K.; Xiao, M. M.; Li, Y.; Zhang, Z. Y.; Zhang, G. J., Rapid and unamplified identification of COVID-19 with morpholino-modified graphene field-effect transistor nanosensor. *Biosens Bioelectron* **2021,** 183, 113206.
6. Woo, C. H.; Jang, S.; Shin, G.; Jung, G. Y.; Lee, J. W., Sensitive fluorescence detection of SARS-CoV-2 RNA in clinical samples via one-pot isothermal ligation and transcription. *Nat Biomed Eng* **2020,** 4, (12), 1168-1179.
7. de Lima, L. F.; Ferreira, A. L.; Torres, M. D. T.; de Araujo, W. R.; de la Fuente-Nunez, C., Minute-scale detection of SARS-CoV-2 using a low-cost biosensor composed of pencil graphite electrodes. *Proc Natl Acad Sci U S A* **2021,** 118, (30).
8. Liu, T.; Hsiung, J.; Zhao, S.; Kost, J.; Sreedhar, D.; Hanson, C. V.; Olson, K.; Keare, D.; Chang, S. T.; Bliden, K. P.; Gurbel, P. A.; Tantry, U. S.; Roche, J.; Press, C.; Boggs, J.; Rodriguez-Soto, J. P.; Montoya, J. G.; Tang, M.; Dai, H., Quantification of antibody avidities and accurate detection of SARS-CoV-2 antibodies in serum and saliva on plasmonic substrates. *Nat Biomed Eng* **2020,** 4, (12), 1188-1196.
9. Deng, J.; Tian, F.; Liu, C.; Liu, Y.; Zhao, S.; Fu, T.; Sun, J.; Tan, W., Rapid One-Step Detection of Viral Particles Using an Aptamer-Based Thermophoretic Assay. *J Am Chem Soc* **2021,** 143, (19), 7261-7266.
10. Lu, M.; Joung, Y.; Jeon, C. S.; Kim, S.; Yong, D.; Jang, H.; Pyun, S. H.; Kang, T.; Choo, J., Dual-mode SERS-based lateral flow assay strips for simultaneous diagnosis of SARS-CoV-2 and influenza a virus. *Nano Converg* **2022,** 9, (1), 39.
11. Tabarov, A.; Vitkin, V.; Andreeva, O.; Shemanaeva, A.; Popov, E.; Dobroslavin, A.; Kurikova, V.; Kuznetsova, O.; Grigorenko, K.; Tzibizov, I.; Kovalev, A.; Savchenko, V.; Zheltuhina, A.; Gorshkov, A.; Danilenko, D., Detection of A and B Influenza Viruses by Surface-Enhanced Raman Scattering Spectroscopy and Machine Learning. *Biosensors (Basel)* **2022,** 12, (12).
12. Li, J.; Lin, R.; Yang, Y.; Zhao, R.; Song, S.; Zhou, Y.; Shi, J.; Wang, L.; Song, H.;

Hao, R., Multichannel Immunosensor Platform for the Rapid Detection of SARS-CoV-2 and Influenza A(H1N1) Virus. *ACS Appl Mater Interfaces* **2021,** 13, (19), 22262-22270.
13. Ahmed, S. R.; Kim, J.; Tran, V. T.; Suzuki, T.; Neethirajan, S.; Lee, J.; Park, E. Y., In situ self-assembly of gold nanoparticles on hydrophilic and hydrophobic substrates for influenza virus-sensing platform. *Sci Rep* **2017,** 7, 44495.
14. Yu, S. T.; Thi Bui, C.; Kim, D. T. H.; A, V. T. N.; Thi Trinh, T. T.; Yeo, S. J., Clinical evaluation of rapid fluorescent diagnostic immunochromatographic test for influenza A virus (H1N1). *Sci Rep* **2018,** 8, (1), 13468.
15. Nguyen, A. V. T.; Dao, T. D.; Trinh, T. T. T.; Choi, D. Y.; Yu, S. T.; Park, H.; Yeo, S. J., Sensitive detection of influenza a virus based on a CdSe/CdS/ZnS quantum dot-linked rapid fluorescent immunochromatographic test. *Biosens Bioelectron* **2020,** 155, 112090.
16. Zhou, H.; Bu, S.; Xu, Y.; Xue, L.; Li, Z.; Hao, Z.; Wan, J.; Tang, F., CRISPR/Cas13a combined with hybridization chain reaction for visual detection of influenza A (H1N1) virus. *Anal Bioanal Chem* **2022,** 414, (29-30), 8437-8445.
17. Xue, L.; Bu, S.; Xu, M.; Wei, J.; Zhou, H.; Xu, Y.; Hao, Z.; Li, Z.; Wan, J., A sensitive fluorescence biosensor based on ligation-transcription and CRISPR/Cas13a-assisted cascade amplification strategies to detect the H1N1 virus. *Anal Bioanal Chem* **2024,** 416, (13), 3195-3203.
